# Supplementary material for: Impairment of Endogenous Synthesis of Omega-3 DHA Exacerbates T-Cell Inflammatory Responses
Source: Int J Mol Sci. 2023 Feb 13;24(4):3717. doi: 10.3390/ijms24043717 (PMC9966148; doi:10.3390/ijms24043717)
Supplement: Supplementary file 1 [file ijms-24-03717-s001.zip › ijms-2195689-supplementary.pdf]

Supplementary Materials:

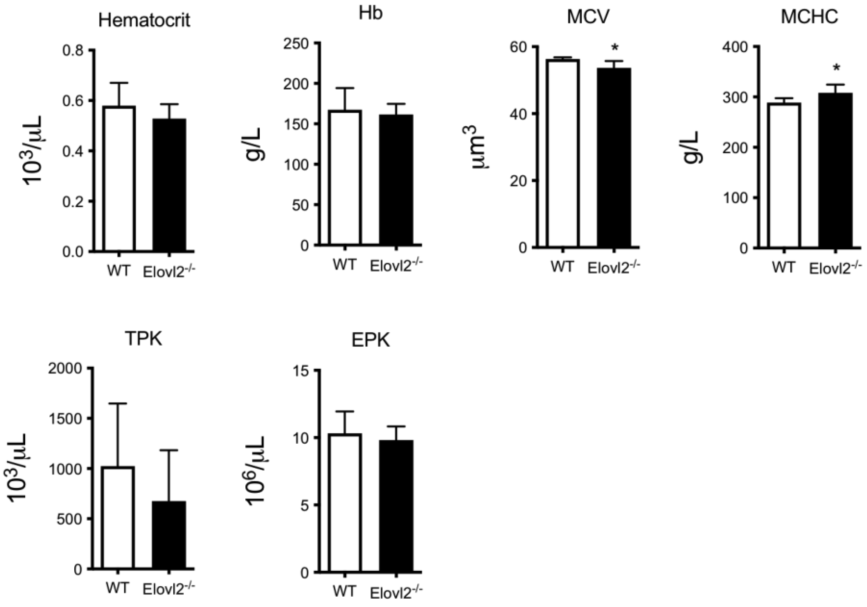

Figure S1. Other clinical parameters in peripheral blood in WT and Elov2$^{-/-}$ mice. * $p < 0.05$.

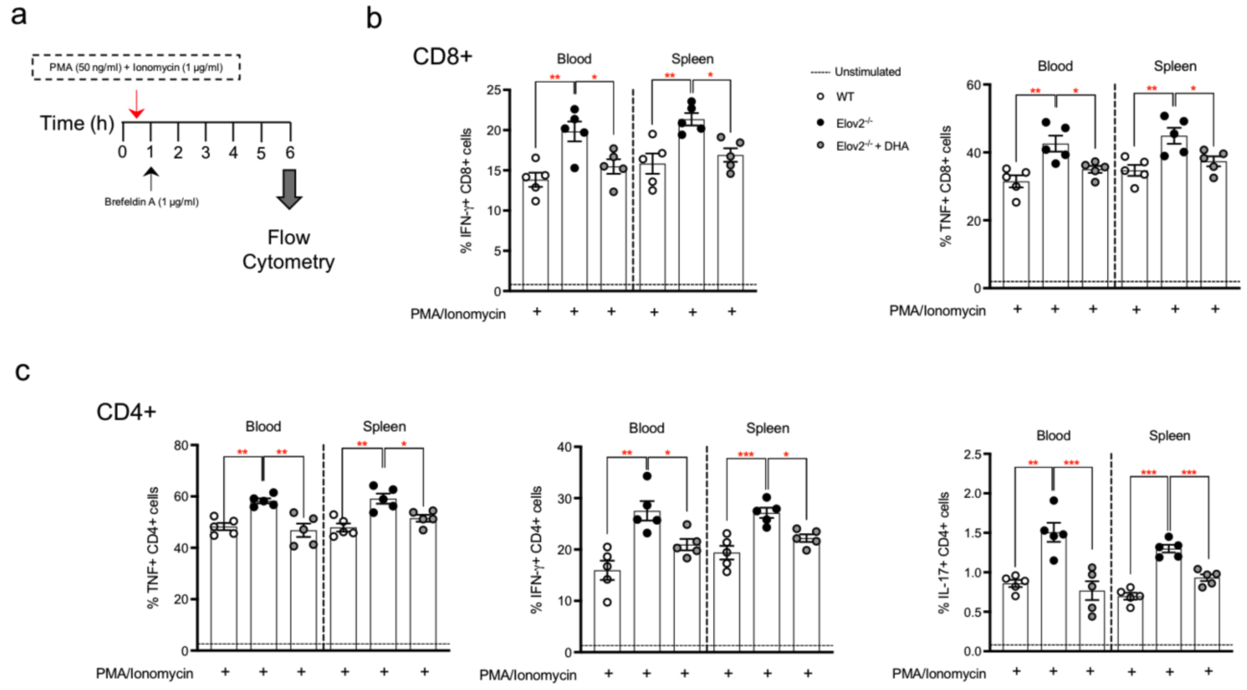

Figure S2. (**a**) Schematic representation of the experimental design; (**b**) histograms of intracellular production of TNF-α and IFN-γ from CD8+ following stimulation with PMA/Ionomycin in blood and spleen of WT, Elov2$^{-/-}$ and Elov2$^{-/-}$ + DHA. (**c**) histograms of intracellular production of IFN-γ and IL-17 from CD4+ following stimulation with PMA/Ionomycin in blood and spleen of WT, Elov2$^{-/-}$ and Elov2$^{-/-}$ + DHA. * $p < 0.05$; ** $p < 0.01$;*** $p < 0.005$.
